# The Influence of Exercise-Associated Small Extracellular Vesicles on Trophoblasts *In Vitro*

**DOI:** 10.3390/biomedicines11030857

**Published:** 2023-03-11

**Authors:** Shuhiba Mohammad, Jayonta Bhattacharjee, Velislava Tzaneva, Kelly Ann Hutchinson, Madeeha Shaikh, Danilo Fernandes da Silva, Dylan Burger, Kristi B. Adamo

**Affiliations:** 1 School of Human Kinetics, Faculty of Health Sciences, University of Ottawa, Ottawa, ON K1N 6N5, Canada; 2 Kidney Research Centre, Department of Cellular and Molecular Medicine, The Ottawa Hospital Research Institute, University of Ottawa, Ottawa, ON K1H 8L6, Canada

**Keywords:** exercise, physical activity, small extracellular vesicles, placenta, trophoblast, pregnancy

## Abstract

Exercise induces the release of small extracellular vesicles (sEVs) into circulation that are postulated to mediate tissue cross-talk during exercise. We previously reported that pregnant individuals released greater levels of sEVs into circulation after exercise compared to matched non-pregnant controls, but their biological functions remain unknown. In this study, sEVs isolated from the plasma of healthy pregnant and non-pregnant participants after a single bout of moderate-intensity exercise were evaluated for their impact on trophoblasts *in vitro*. Exercise-associated sEVs were found localized within the cytoplasm of BeWo choriocarcinoma cells, used to model trophoblasts in vitro. Exposure to exercise-associated sEVs did not significantly alter BeWo cell proliferation, gene expression of angiogenic growth factors *VEGF* and *PLGF*, or the release of the hormone human chorionic gonadotropin. The results from this pilot study support that exercise-associated sEVs could interact with trophoblasts in vitro, and warrant further investigation to reveal their potential role in communicating the effects of exercise to the maternal–fetal interface.

## 1. Introduction

Exercise during pregnancy is well known to bestow benefits on both mother and fetus, potentially improving health across two generations. To promote maternal health and as a front-line therapy for mitigating the risk of pregnancy disorders, the 2019 Canadian Guideline for Physical Activity throughout Pregnancy recommends that those without contraindications should engage in at least 150 min of moderate-intensity physical activity per week [1]. Maternal physical activity is associated with decreased risk of pregnancy complications, including preeclampsia, gestational diabetes mellitus, and gestational hypertension [2], and improvement of prenatal depressive symptoms [3] while reducing blood glucose levels [4]. Prenatal physical activity is also associated with beneficial neonatal outcomes such as reduced odds of macrosomia [5] and fat mass at birth [6]. While the advantages are numerous and well established, the biological mechanisms by which these benefits are communicated to the mother and fetus are not well understood.

Exercise has been posited to alter the structure and physiology of the placenta [7], the critical interface between mother and fetus chiefly responsible for maternal–fetal communication. The placenta supports fetal growth and survival by mediating the exchange of gases, nutrients, and waste, while providing endocrine and immune support. As a transient discoid organ anchored to the maternal uterus, the placenta is composed of a heterogeneous population of cells organized in a manner to ensure sufficient maternal–fetal exchange between the juxtaposed maternal and fetal circulatory systems. In the placenta, maternal exercise has been shown to impact oxidative stress [8], nutrient transporters [9,10], and angiogenic growth factors [11,12]. Data from exercise interventions also show beneficial effects on placental growth [13] and volume available for maternal–fetal exchange [14,15]. While it is evident that exercise impacts placental function, the biological mechanisms contributing to these changes are less clear.

The benefits of exercise are hypothesized to be mediated in part by the release of bioactive molecules into circulation after exercise, including myokines (cytokines produced and released by skeletal muscle) [16,17] and small extracellular vesicles (sEVs) [18,19]. sEVs (historically referred to as “exosomes” [20]) are lipid membrane-enclosed particles (~20–120 nm in diameter) secreted by cells into extracellular space and contain bioactive compounds such as nucleic acids (i.e., mRNAs and miRNAs), lipids, and proteins [21,22]. sEVs are theorized to facilitate long-distance intercellular communication by interaction or transfer of their biological cargo from donor to target cells [23,24]. Data from both human and animal studies demonstrate that sEVs are released into circulation after exercise (reviewed by [19]) and contain biological molecules potentially involved in tissue cross-talk in response to exercise [18]. We previously reported that pregnant individuals release greater levels of sEVs into circulation compared to non-pregnant individuals after a single bout of moderate-intensity exercise [25]. The potential biological function and cellular targets of circulating sEVs released in response to maternal exercise are unknown. Since the placenta can release and take up sEVs as a part of maternal–fetal communication [26,27], exercise-associated sEVs could possibly act on the placenta. Therefore, circulating sEVs may represent a potential mechanism involved in improving placental function in the context of maternal exercise.

In this study, we examined in pilot experiments whether exposure to circulating sEVs obtained after acute exercise affected trophoblasts *in vitro*. Trophoblast cells are specialized epithelial cells that constitute the maternal–fetal interface and are responsible for hormone production and the exchange of gases, nutrients, and waste between mother and fetus. First, we determined whether circulating exercise-associated sEVs from healthy pregnant and non-pregnant individuals could interact with trophoblasts *in vitro*. Further, we evaluated whether exposure to exercise-associated plasma sEVs could influence metrics of trophoblast biology, including proliferation, expression of angiogenic growth factors known to be altered in the placenta of physically active vs. inactive mothers [11], and secretion of the major pregnancy hormone, human chorionic gonadotropin (b-hCG). Treatment with sEVs from healthy non-pregnant controls was used to determine whether the potential effects on trophoblast biology were associated with pregnancy status or exercise stimulus. In this set of pilot experiments, we hypothesized that exercise-associated sEVs from pregnant individuals would elicit greater effects on trophoblast biology compared to exercise-associated sEVs from non-pregnant controls. 

## 2. Materials and Methods

### 2.1. Ethical Approval and Study Participants

Experimental procedures were approved by the University of Ottawa Research Ethics Board (file number: H-06-18-634). All protocols were performed in fulfillment of the guidelines described in the Declaration of Helsinki. Informed written consent was secured from participants after an explanation of the study procedures. Pregnant and non-pregnant individuals were recruited from the Ottawa area (ON, Canada) for participation in the study. Healthy individuals without contraindication to exercise between the ages of 18 and 40 were eligible for inclusion, with a self-reported pre- or non-pregnant body mass index (BMI) of 18.5–29.9 kg/m^2^. Participants were required to be weight-stable (±5 kg) for approximately six months before the study. Pregnant participants needed to be between 13 and 28 weeks gestation carrying a singleton fetus to participate in the study. Those with chronic health conditions including hypertension, diabetes (pre/non-pregnant or gestational diabetes), and untreated thyroid disease and frequent users of tobacco, drugs, or alcohol were excluded from participation.

### 2.2. Acute Exercise Procedure

The acute exercise stimulus followed procedures outlined by Hutchinson et al. (2019) [28] and Mohammad et al. (2021) [25]. Briefly, participants were requested to refrain from exercise and food for 8 h before the acute exercise session. Participants were provided with a standardized snack, after which resting heart rate was determined as previously described [25,28]. A target range of 40–59% of heart rate (HR) reserve was used to specify moderate-intensity exercise [29,30], where HR reserve was calculated using the Karvonen equation [31] as described previously [25,28]. The moderate-intensity acute exercise session consisted of a brisk 30 min treadmill walk with continuous HR monitoring using a Polar V800 HR monitor (Polar Electro, Lachine, QC, Canada). A short warm-up (3 min at 2% incline at a speed of 2 miles per hour (mph)) was followed by an incremental phase (6% incline), where treadmill speed was increased by 0.2 mph every minute until the upper range of moderate-intensity HR reserve (59%) was met. Once this range was met, the participants continued to exercise for 30 min. Blood was collected (10 mL) immediately before (at rest) and after the exercise session from the median cubital vein using potassium EDTA blood collection tubes (#367863; BD Biosciences, Mississauga, ON, Canada). Plasma was promptly processed by centrifugation at 1700× *g* for 15 min at 4 °C, and samples were stored at −80 °C until further analyses.

### 2.3. sEV Isolation and Labeling

Isolation of sEVs from plasma was performed using differential ultracentrifugation as described previously [25,32,33]. Plasma samples (1.0 mL) were thawed rapidly at 37 °C, then kept on ice for all remaining procedures. Samples were first centrifuged at 20,000× *g* for 20 min at 4 °C to remove large EVs and apoptotic bodies. The remaining supernatant was centrifuged at a speed of 100,000× *g* using a Beckman Coulter Optima MAX ultracentrifuge (Beckman Coulter Inc., Brea, CA, USA) equipped with a TLA-55 rotor (Beckman Coulter) for 90 min at 4 °C. The resulting pellet of sEVs was washed with 1.0 mL of 0.1 µm filtered phosphate-buffered saline (PBS) and then centrifuged at 100,000× *g* as described above. The final residual pellet of sEVs was resuspended in 100 µL of 0.1 µm filtered PBS. Aliquots of 10 µL of this suspension were separated and frozen at −80 °C for further analysis. One 10 µL aliquot was used for protein extraction and quantification to standardize sEV treatment concentrations for subsequent functional assays. To extract protein, 1 µL of 10× radioimmunoprecipitation assay (RIPA) buffer with protease inhibitor cocktail (MilliporeSigma Canada Co., #P8340, Oakville, ON, Canada) was added to a 10 µL sEV aliquot. Samples were sonicated using a bath sonicator for 1 min to achieve EV and protein lysis and subsequently were incubated on ice for 30 min before protein quantification using a DC protein assay (Bio-Rad Laboratories, #5000112, Mississauga, ON, Canada). sEVs from this cohort were previously validated to be the expected size (~100–120 nm) and displayed characteristics confirming the presence of these particles (i.e., expression of classical sEV protein markers TSG-101 and flotillin-1, absence of non-sEV marker calnexin, and intact membrane integrity and characteristic size as determined by transmission electron microscopy [25]. Where indicated, sEVs were fluorescently labeled with PKH26, a lipophilic membrane dye, using the PKH26 Red Fluorescent Cell Linker Kit according to the manufacturer’s instructions (Phanos Technologies, MilliporeSigma Canada Co., #MINI26-1KT). Following the incorporation of the dye, the labeled sEVs were centrifuged at 100,000× *g* for 90 min at 4 °C, and the resulting sEV pellets were resuspended in 100 µL of 0.1 µm filtered PBS and stored at −80 °C for subsequent sEV internalization and interaction fluorescence assays.

### 2.4. Cell Culture

BeWo choriocarcinoma cells were obtained from the American Type Culture Collection (ATCC CCL-98, Manassas, VA, USA) and grown in Ham’s F-12K (Kaighn’s) medium (Gibco, Thermo Fisher Scientific, #21127022, Waltham, MA, USA) supplemented with 10% fetal bovine serum (FBS) and incubated at 37 °C and 5% CO_2_ in a humidified environment. For all sEV treatment experiments, cells from passages 7–12 were used in the presence of F-12K medium supplemented with 10% EV-depleted FBS. EV-depletion of undiluted FBS was achieved by ultracentrifugation at 100,000× *g* for 90 min at 4 °C [34] and retention of the resulting supernatant, confirmed by nanoparticle tracking analysis. Cells were experimentally manipulated 48 h post-seeding.

### 2.5. sEV Localization by Fluorescence Confocal Microscopy

BeWo cells (2.5 × 10^4^) were plated in 8-well chamber slides (ibidi USA, #80826, Fitchburg, WI, USA) and incubated with 2.5 µg/mL of PKH26-labeled sEVs (or PBS control) overnight (16 h) at 37 °C and 5% CO_2_. The cell culture supernatant was removed, and wells were vigorously washed five times with PBST and then fixed with 10% buffered formalin for 10 min at room temperature. All subsequent steps were conducted at room temperature with three PBST washes between each step unless noted otherwise. Fixative was removed, and then cells were permeabilized using 0.1% Triton X-100 in PBS for 5 min. Next, slides were incubated with phalloidin-iFluor 488 Reagent (1:1000; Abcam Inc., #ab176753, Cambridge, MA, USA) in 1% BSA in PBS for 25 min. Finally, 1 drop of NucBlue Fixed Cell ReadyProbes Reagent (DAPI) (Invitrogen, Thermo Fisher Scientific, #R37606) was added to each well for 5 min. Mounting media (ibidi USA, #500001) were added to each well before imaging using an inverted Zeiss LSM 880 AxioObserver Z1 laser-scanning confocal microscope with Airyscan FAST detector equipped with Zen Black software (version 2.3, Carl Zeiss Microscopy GmbH, Jena, Germany). Images were taken using a 63× oil-immersion objective lens (Carl Zeiss Microscopy GmbH, Plan Apochromat 63/1.4 NA oil) with optical slices (z-stacks) at a thickness of 0.20 µm. The confocal was equipped with lasers emitting at 405, 488, and 561 nm which were used for the excitation of each fluorophore: DAPI (Ex/Em 405/450 nm), phalloidin (Ex/Em 488/516 nm), and PKH26 (Ex/Em 561/579 nm). Each confocal microscopy image was acquired using the same imaging parameters. Images were subjected to linear unmixing of the measured spectral profiles for each fluorophore (DAPI, phalloidin, and PKH26) using Zen Black software (version 2.3) to account for signal crossover between spectral channels. Representative maximum intensity projections were acquired from a subset of z-stacks corresponding to the middle of the cells. For each condition, a minimum of three random fields of view were selected and examined for sEV localization. To increase the quality of the images for display purposes, lookup tables for the phalloidin channel image were set to “Magenta” and for the PKH26 channel image were set to “Green” using Fiji software (version 2.3.0/1.53f, U.S. National Institutes of Health, Bethesda, Maryland, USA). 

For the localization experiments, a range of PKH26-labeled sEV concentrations was initially tested (1, 2.5, 5, and 10 µg/mL) for visualization by confocal microscopy. The concentration of 2.5 µg/mL of sEVs incubated overnight (16 h) was found to be the exposure with the best signal-to-noise ratio. 

### 2.6. Proliferation Assessment by Ki67 Immunostaining

To assess the influence of exercise-associated plasma sEVs on BeWo cell proliferation in vitro, Ki67 immunostaining was used. BeWo cells (2.5 × 10^4^) were seeded onto 8-well chamber slides (ibidi USA, #80826) and incubated with 10 µg/mL sEVs (or PBS control) for 24 h at 37 °C and 5% CO_2_ in duplicate. Then, the cell culture supernatant was removed and wells were washed three times with PBST. All of the following steps were carried out at room temperature unless stated otherwise. Cells were fixed with 10% formalin for 10 min and then washed three times with PBST. Cells were then permeabilized using 0.1% Triton X-100 in PBS for 5 min followed by three washes with PBST. Cells were blocked for 30 min with BlockAid Blocking Solution (Invitrogen, Thermo Fisher Scientific, #B10710) and then incubated with recombinant anti-Ki67 rabbit monoclonal antibody (SP6) (1:250; Abcam Inc, #ab16667) at 4 °C in PBST overnight. Negative controls omitted the primary antibody. The following day, wells were washed three times with PBST and then incubated with goat AlexaFluor 488 anti-rabbit IgG (H+L) Superclonal recombinant secondary antibody (1:1000; Invitrogen, Thermo Fisher Scientific, #A27034) in PBST for 60 min. Wells were washed three times with PBST and then incubated with 1 drop of NucBlue Fixed Cell ReadyProbes Reagent (DAPI) (Invitrogen, Thermo Fisher Scientific, #R37606) per well for 5 min. Mounting media (ibidi USA, #500001) were added to each well before imaging using a ThermoFisher FL Auto 2 inverted automated epifluorescent microscope equipped with version Auto2 software (Invitrogen, Thermo Fisher Scientific). Each well was divided into four quadrants, with one image taken at 20× magnification per quadrant for a total of four images per well. All experiments were performed in triplicate. A ratio of Ki67 immunostaining intensity to nuclear area was measured using Fiji software (version 2.3.0/1.53f, U.S. National Institutes of Health). 

### 2.7. RNA Isolation and Quantitative Real-Time Polymerase Chain Reaction (qPCR)

BeWo cells were seeded at a density of 1.0 × 10^5^ in 12-well dishes; 48 h later, they were treated with 10 µg/mL plasma sEVs (or PBS control) for 24 h at 37 °C and 5% CO_2_. Cell culture supernatant was collected and stored at −80 °C for downstream b-hCG analysis described below. Cells were washed twice with cold sterile PBS and then lysed for total RNA isolation using an Illustra RNAspin Mini isolation kit (Cytiva Life Sciences, Fisher Scientific Company, #25050071, Ottawa, ON, Canada) as per the manufacturer’s instructions. Isolated total RNA was eluted in RNase-free water and was analyzed for concentration and purity using spectrophotometry (Take3, Gen5 software version 1.11.5, BioTek Instruments Inc., Winooski, VT, USA). RNA integrity was verified using a 2% agarose gel stained with SYBR Safe DNA gel stain (Invitrogen, Thermo Fisher Scientific, #S33102) and electrophoresis of bromophenol blue-labeled RNA aliquots at 100 V for 30 min in TAE (Tris-acetate-EDTA) buffer. RNA bands were visualized by ultraviolet transillumination using a ChemiDoc XRS+ system (Bio-Rad Laboratories, Mississauga, ON, Canada). Then, 0.5 µg of RNA was reverse transcribed into cDNA using an iScript cDNA synthesis kit (Bio-Rad Laboratories, #1708891) and a Biometra TPersonal Combi Thermocycler (Analytik Jena, Jena, Germany) according to the manufacturer’s protocol, and then stored at −20 °C until further analysis. A total of 25 ng of cDNA was used for quantitative polymerase chain reaction (qPCR) using TaqMan Advanced Master Mix (Applied Biosystems, Thermo Fisher Scientific, #444557). Samples were loaded into a Rotor-Gene 3000 real-time DNA detection system with Rotor-Gene software (version 6.1.93, Corbett Research, Sydney, Australia). The expression of angiogenic growth factors *VEGF* and *PLGF* was measured relative to an endogenous control, *GAPDH*. Taqman gene expression assay probes labeled with 6-FAM (6-carboxyfluorescein) fluorescent dye were used for the detection of *VEGF* (Hs00900055_m1), *PLGF* (Hs00182176_m1), and *GAPDH* (Hs02786624_g1). The qPCR reaction was as follows: hold at 50 °C for 2 min, then hold at 95 °C for 2 min, followed by 40 cycles consisting of denaturing at 95 °C for 3 s and hold at 60 °C for 30 s. All qPCR reactions were performed in duplicate with appropriate controls (no cDNA reverse transcriptase cDNA control and no template qPCR control), and all experiments were conducted in triplicate. Corresponding threshold cycle (CT) values were recorded, and relative gene expression was calculated using the 2^−ΔΔCT^ method [35]. Data from the target genes (*VEGF* and *PLGF*) were expressed as a ratio to *GAPDH* gene expression for normalization. Gene expression values from PBS control treatment conditions were considered as control. 

### 2.8. b-hCG Assay

Cell culture supernatant collected from the gene expression analyses described above were used to determine the effect of sEV treatment on b-hCG production in BeWo cells after 24 h of exposure using a DRG b-hCG ELISA kit (DRG International, #EIA-1911, Springfield, NJ, USA) as per the manufacturer’s instructions. b-hCG concentration was normalized to mg of total RNA isolated from the corresponding gene expression experiments and is presented in mIU/mL.

### 2.9. Statistical Analysis

All data are presented as mean ± standard deviation (SD) from three independent experiments. All statistical analyses and graphs were generated using GraphPad Prism version 9.3 (GraphPad Software, La Jolla, CA, USA). A one-way ANOVA with Tukey’s post-test (if applicable) was used to compare whether no treatment (PBS control) was different from exposure to exercise-associated sEVs from pregnant and non-pregnant individuals. The homogeneity assumption was confirmed by Levene’s test. Statistical significance was considered when *p* < 0.05.

## 3. Results

### 3.1. Exercise-Associated sEVs Interact with BeWo Cells

**Figure 1:**
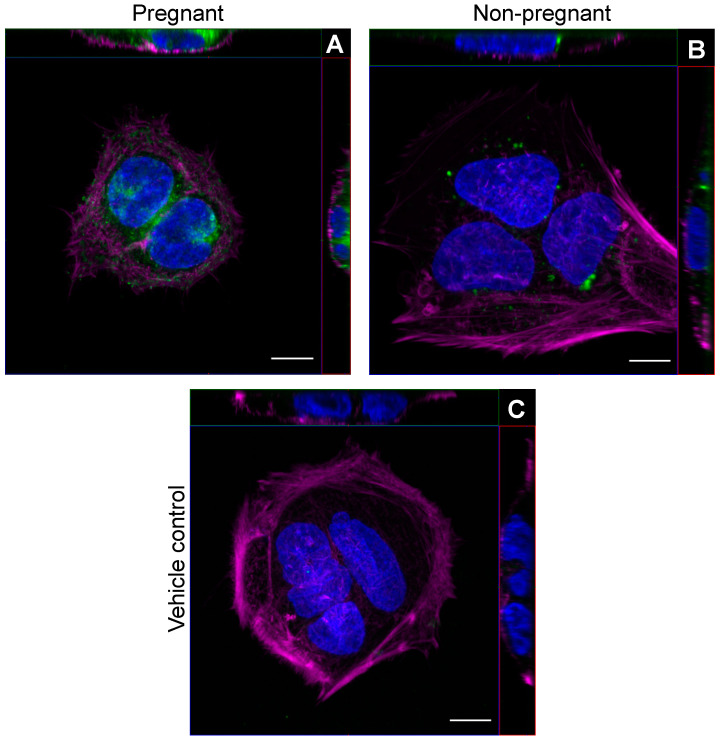
Exercise-associated sEVs were found within BeWo cells *in vitro*. Representative confocal microscopy images of BeWo cells after overnight (16 h) incubation with 2.5 µg/mL PKH26-labeled sEVs from pregnant (**A**) and non-pregnant (**B**) plasma post-exercise. Panel (**C**) shows a representative vehicle control image (PBS). Blue represents DAPI staining for nuclei, magenta depicts phalloidin staining, and green shows PKH26-labeled sEVs. “Magenta” and “Green” lookup tables were used to display phalloidin and sEV labeling, respectively. For each panel, orthogonal projections show the XY (main image), YZ (right of main image), and XZ (top image) planes. All images were taken using a 63× objective lens with oil immersion. Scale bar = 10 µm for all images.

We first examined whether circulating sEVs obtained from pregnant and non-pregnant individuals after a bout of acute moderate-intensity exercise could be found within trophoblast-like cells *in vitro.* Representative images obtained by confocal microscopy show that PKH26-labeled sEVs from both pregnant and non-pregnant individuals obtained post-exercise localized within the cytoplasm of BeWo cells after overnight incubation (Figure 1). 

### 3.2. BeWo Cell Proliferation Was Not Affected upon Exposure to Exercise-Associated sEVs

**Figure 2:**
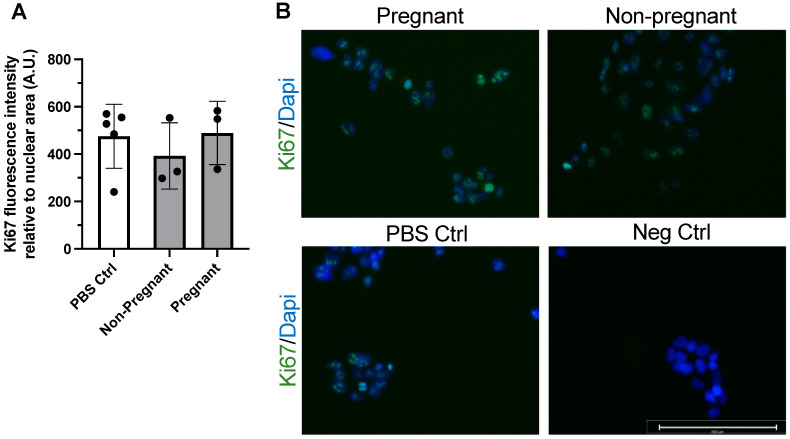
Exercise-associated sEVs did not affect BeWo cell proliferation. There were no differences in proliferation when no treatment (PBS Ctrl) was compared to treatment with exercise-associated sEVs from pregnant and non-pregnant individuals (F = 0.469, *p* = 0.642) (**A**). Representative merged fluorescence images of each condition (**B**) and a negative control (Neg Ctrl) where primary antibody was omitted are shown, where blue depicts DAPI staining for nuclei and green shows Ki67 positive signal. All experiments were conducted in triplicate, with sEVs obtained from *n* = 3 pregnant and *n* = 3 non-pregnant participants, with corresponding PBS controls (*n* = 5). Scale bar = 200 µm. Neg ctrl, negative control; PBS Ctrl, phosphate-buffered saline control.

Immunostaining of the protein Ki67 was used to assess proliferation in BeWo cells after 24 h incubation with exercise-associated sEVs. Ki67 is a widely used marker of cell proliferation and has been used in the examination of trophoblast and BeWo cell proliferation and phenotype [36]. Exposure to sEVs obtained from pregnant participants and non-pregnant controls after exercise did not influence BeWo cell proliferation after 24 h when compared to vehicle treatment (PBS control) (Figure 2). 

### 3.3. Exposure to Plasma sEVs Did Not Alter the Gene Expression of Angiogenic Growth Factors in BeWo Cells 

**Figure 3:**
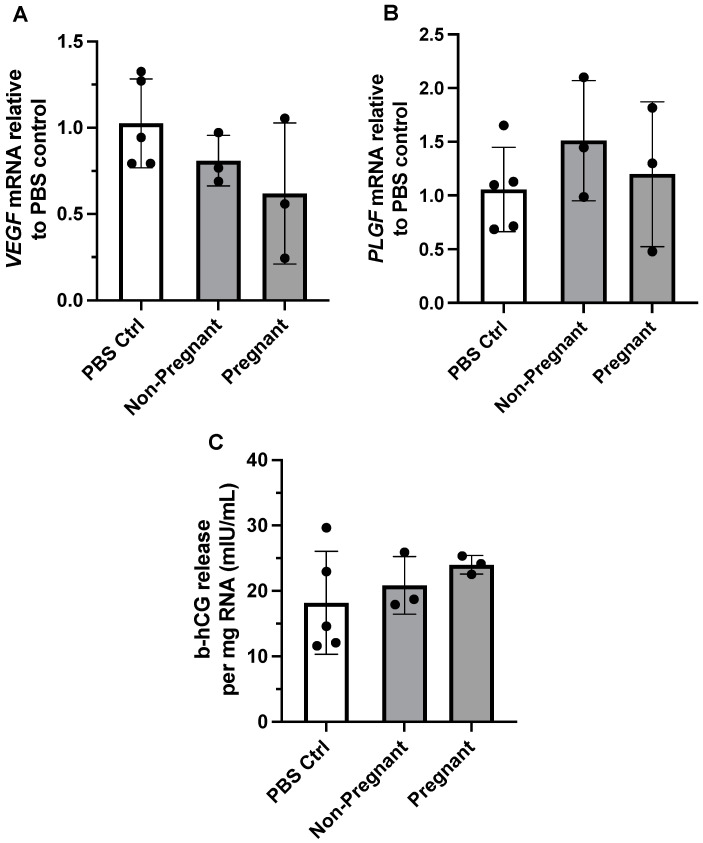
The effect of exercise-associated sEVs on relative gene expression of angiogenic growth factors and b-hCG production in BeWo cells. Gene expression of *VEGF* (**A**) and *PLGF* (**B**) was not altered upon exposure to circulating exercise-associated sEVs (10 µg/mL for 24 h) obtained from pregnant and non-pregnant individuals compared to PBS control (PBS Ctrl) treatment (F = 1.98, *p* = 0.200, and F = 0.726, *p* = 0.513, respectively). (**C**) There were no differences in b-hCG cell media levels when no treatment was compared to treatment with exercise-associated sEVs from pregnant and non-pregnant participants (F = 0.885, *p* = 0.450). All experiments were conducted in triplicate, with sEVs obtained from *n* = 3 pregnant and *n* = 3 non-pregnant participants, with corresponding PBS controls (*n* = 5).

We previously reported that maternal exercise was associated with differential expression of angiogenic proteins in the placenta of individuals categorized as physically active vs. inactive during pregnancy [11]. We therefore determined whether exposure to circulating exercise-associated sEVs from pregnant or non-pregnant individuals affected gene expression of *VEGF* and *PLGF* in BeWo cells. Gene expression of both *VEGF* and *PLGF* did not differ between treatment with PBS control vs. exercise-associated sEVs obtained from both groups (F = 1.98, *p* = 0.200, and F = 0.726, *p* = 0.513, respectively) (Figure 3A,B). 

### 3.4. Human Chorionic Gonadotropin Levels Were Not Affected upon Exposure to Exercise-Associated sEVs

The hormone b-hCG is known to be produced and released from BeWo cells *in vitro* in the process of syncytialization, an essential part of trophoblast differentiation and function [37,38]. We examined the levels of b-hCG in cell media after incubation with exercise-associated plasma sEVs from pregnant and non-pregnant participants. Exposure to exercise-associated sEVs did not result in differing levels of b-hCG in cell media when compared to no treatment (PBS control exposure) (F = 0.885, *p* = 0.450; Figure 3C).

## 4. Discussion

This study aimed to determine whether sEVs released after a single bout of acute moderate-intensity exercise in pregnant individuals and respective non-pregnant controls can be internalized by cells modeling placental trophoblasts *in vitro* and alter their function. We provide the first evidence that sEVs released after exercise into circulation could interact with trophoblasts *in vitro* but do not alter traditional indices of trophoblast biology, including proliferation, gene expression of angiogenic markers, or production of the pregnancy hormone b-hCG. sEVs are postulated to be an important delivery mechanism in the adaptive response to exercise on a systemic level [18,19,39]. This study provides preliminary evidence that may facilitate our understanding of how exercise is communicated from the mother to the fetus during pregnancy via the maternal–fetal interface.

Central to the hypothesis that sEVs communicate the benefits of exercise systemically is their uptake into recipient tissues and cells, or surface interactions with activating receptors. Exercise-associated sEVs from both pregnant and non-pregnant individuals were localized within BeWo cells. Few studies have evaluated whether sEVs released into circulation in response to exercise can be taken up by or interact with recipient cells. Notably, Whitham et al. (2018) showed that fluorescently labeled EVs from mouse myoblasts could be localized within mouse liver hepatocytes and that cargo of plasma EVs from exercising mice was incorporated into mouse liver hepatocytes [18]. In the context of exercise training and cardiomyocyte injury, PKH26-labeled EVs isolated from the plasma of exercising rats were found to be internalized into cardiomyocytes in vitro after a 6 h incubation period [40]. In a study conducted by Just et al. (2020), sEVs released after acute blood flow-restricted resistance exercise in healthy young men were localized within muscle stem cells and fibro-adipogenic progenitor cells in vitro [41]. Our proof-of-concept experiments show that exercise-associated sEVs can also interact with trophoblasts, specialized cells that are in direct contact with maternal blood and constitute the maternal–fetal interface. Since evidence suggests that EV uptake is not a passive process and involves a variety of energy-requiring endocytic pathways (reviewed by [42]), we postulate that exercise-associated sEVs may play a role in modifying trophoblast function in the context of maternal exercise. 

Having observed that exercise-associated sEVs could interact with trophoblast-like cells *in vitro*, we sought to investigate their potential impact on trophoblast physiology. The secretion of b-hCG in cell culture media was investigated as a marker of BeWo cell and trophoblast differentiation. Impaired trophoblast differentiation and fusion are seen in pregnancy pathologies including pre-eclampsia [38], for which risk is mitigated by regular engagement in physical activity during pregnancy [2]. The release of b-hCG was not affected by exposure to exercise-associated sEVs from pregnant or non-pregnant individuals, nor was BeWo cell proliferation. Trophoblasts produce VEGF and PlGF to promote branching and non-branching placental angiogenesis, respectively [43,44]. Given that we previously reported differential expression of angiogenic growth factors VEGF, PlGF, and their respective receptors in term placenta of individuals categorized physically active or inactive [11], we aimed to determine whether gene expression of *VEGF* or *PLGF* was affected by exercise-associated sEVs. Regardless of pregnancy status, exposure to exercise-associated sEVs did not produce changes in *VEGF* or *PLGF* expression levels in BeWo cells. Our observations differ from data in human umbilical vein endothelial cells (HUVECs), where exposure to plasma sEVs isolated from pregnant and non-pregnant individuals at resting conditions was found to increase endothelial cell migration similarly to VEGF-induced migration [27]. It must be noted that Salomon et al. (2014) [27] used a concentration of sEVs that was 10-fold higher than concentrations used to expose trophoblasts in our study (i.e., 100 µg/mL vs. 10 µg/mL EV protein, respectively), and they did not examine gene expression of *VEGF*.

Relatively few studies to date have determined the physiological impact of exercise-associated sEVs on the biological functions of target cells, and none have been conducted on trophoblasts or cells constituting the maternal–fetal interface. The majority of work on the physiological consequences of exercise-associated sEVs involve animal models, where their interactions have been shown to delay prostate cancer progression [45], provide sustained cardioprotection [40], and elicit beneficial effects in ischemic stroke [46]. In humans, sEVs obtained after blood flow-restricted resistance exercise in healthy men were found to increase the proliferation of fibro-adipogenic progenitor cells [41]. The lack of biological impact on trophoblast biology demonstrated in the present study is likely due to a variety of factors. The bioactivity of sEVs is largely dependent on their diverse biological cargo, which is influenced by the cells of origin. Identifying the contents and origins of exercise-associated sEVs in pregnant and non-pregnant participants was beyond the scope of this preliminary study but represents a critical knowledge gap in this emerging field. Characterization of the biological contents and cellular origins of exercise-associated maternal sEVs will allow for the development of more targeted hypotheses regarding assessments of trophoblastic function. Exercise training status may influence the biological contents of exercise-associated sEVs, as Nair et al. (2020) reported that microRNA profiles differed in circulating sEVs obtained from sedentary vs. active older men [47]. In the present study, we were unable to objectively validate the habitual physical activity patterns of the study participants. Future studies should examine whether exercise training or chronic habitual exercise could alter the bioactivity of maternal sEVs, and evaluate their potential impact on trophoblasts. Currently, there is inconsistent evidence to suggest that exercise intensity or modality could affect sEV release and contents [19,48,49,50]. It is unknown whether differing exercise intensities (i.e., moderate vs. vigorous intensity) could impact sEV cargo, and whether a specific intensity or threshold is required to produce functional changes in trophoblasts. 

Our study presents some strengths and limitations. As noted by others, caution must be exercised when extrapolating results obtained using transformed immortalized cell lines, including BeWo choriocarcinoma cells, in the modeling of normal trophoblast populations [51,52,53]. In this case, while not an exact simulation of trophoblasts, readily available and accessible BeWo cells provide invaluable insights into trophoblast function [52,53,54]. Future experiments deducing the potential function of sEVs in the context of maternal exercise should employ primary human trophoblasts or trophoblast populations obtained from the derivation and differentiation of trophoblast stem cells [55,56]. Another limitation relates to pitfalls associated with physical-based sEV isolation methods including differential ultracentrifugation. Lipoproteins have been reported to be isolated alongside sEVs using physical-based isolation methods, leading to the contamination of sEV isolate fractions [57,58]. Lipoproteins may also carry bioactive molecules such as miRNAs [59,60], which may have introduced potential confounders in the current preliminary study. Future studies should employ alternative sEV isolation methods (i.e., combinations of density gradient centrifugation and size-exclusion chromatography) to minimize co-isolation of contaminating non-sEV species. Further, co-isolated lipoproteins may compete with sEVs upon labeling with lipid-anchored fluorophores, including PKH26 [61,62,63]. Therefore, future investigations should characterize the extent to which samples may be contaminated with co-isolates that could result in the unintended labeling of non-sEV targets in uptake studies.

A strength of the sEV localization analysis stems from the use of an Airyscan detector in conjunction with confocal microscopy allowing for enhanced detection not available in traditional confocal systems [64]. However, it is important to note that the confocal imaging studies presented here are qualitative in nature. Another strength is the duration and type of exercise selected for the representation of acute exercise during pregnancy. The Canadian evidence-based physical activity guidelines throughout pregnancy recommend individuals engage in a minimum of 150 min of moderate-intensity physical activity to achieve benefits, within a recommended heart rate range based on age [1]. We therefore intentionally designed a physiologically appropriate exercise session for pregnancy, while the majority of studies to date on exercise-associated sEVs involve sustained vigorous-intensity exercise until volitional exhaustion in men (reviewed by [19]). Low sample size was a major limitation of our study, but herein we provide pilot data to support the continued exploration of maternal exercise-associated sEVs and their potential impact on placental function. 

## 5. Conclusions

In summary, our preliminary results show that exercise-associated sEVs could be localized within trophoblast-like cells *in vitro*. Whether they can produce biological changes sufficient to improve trophoblast biology or stimulate other intercellular signaling pathways that may transmit signals to the fetus remain unknown. Since the placenta is primarily responsible for maternal–fetal communication and fetal growth, further investigation is warranted to determine the biological impact and mechanisms linking maternal sEVs to the benefits sustained from engagement in exercise during pregnancy.

## Data Availability

Data are available from the corresponding author upon reasonable request.

## Sample Availability

Samples from this study are not available.
